# Supplementary material for: COZOID: contact zone identifier for visual analysis of protein-protein interactions
Source: BMC Bioinformatics. 2018 Apr 6;19:125. doi: 10.1186/s12859-018-2113-6 (PMC5889581; doi:10.1186/s12859-018-2113-6)
Supplement: Supplementary file 2 — Software build. Executable binary file of the software tool. (ZIP 118,784 kb) [file 12859_2018_2113_MOESM2_ESM.zip › cozoid/java/docs/beansbinding-1.2.1-doc.zip!/org/jdesktop/swingbinding/JListBinding.html]

JListBinding (Beans Binding)


---


|  |  |  |
| --- | --- | --- |
| **Overview** \| **Package** \| **Class** \| **Use** \| **Tree** \| **Deprecated** \| **Index** \| **Help** | |  |
| **PREV CLASS**   **NEXT CLASS** | **FRAMES**    **NO FRAMES**     **All Classes** |  |
| SUMMARY: NESTED \| FIELD \| CONSTR \| METHOD | DETAIL: FIELD \| CONSTR \| METHOD |  |


---


## org.jdesktop.swingbinding Class JListBinding<E,SS,TS>

```
java.lang.Object
  org.jdesktop.beansbinding.Binding<SS,SV,TS,TV>
      org.jdesktop.beansbinding.AutoBinding<SS,java.util.List<E>,TS,java.util.List>
          org.jdesktop.swingbinding.JListBinding<E,SS,TS>
```

**Type Parameters:**: `E` - the type of elements in the source `List`: `SS` - the type of source object (on which the source property resolves to `List`): `TS` - the type of target object (on which the target property resolves to `JList`)

---

``` public final class JListBinding<E,SS,TS> extends AutoBinding<SS,java.util.List<E>,TS,java.util.List> ```

Binds a `List` of objects to act as the elements of a `JList`.
Each object in the source `List` provides one element in the `JList`.
By setting a `DetailBinding`
you can specify the property to use to derive each list element from its
corresponding object in the source `List`. The default `DetailBinding` uses
the objects directly. Instances of `JListBinding` are obtained by
calling one of the `createJListBinding` methods in the `SwingBindings`
class.

Here is an example of creating a binding from a `List` of `Person`
objects to a `JList`:

```
    // create the person list
    List people = createPersonList();

    // create the binding from List to JList
    JListBinding lb = SwingBindings.createJListBinding(READ, people, jList);

    // define the property to be used to derive list elements
    ELProperty fullNameP = ELProperty.create("${firstName} ${lastName}");

    // add the detail binding
    lb.setDetailBinding(fullNameP);

    // realize the binding
    lb.bind();
```

The `JList` target of a `JListBinding` acts as a live view of
the objects in the source `List`, regardless of the update strategy (the
meaning of the update strategy is clarified later
in this document). `JListBinding` listens to the property specified for
any `DetailBinding`, for all objects in the `List`, and updates
the values displayed in the `JList` in response to change. If the
`List` is an instance of `ObservableList`, then changes to the
`List` contents (such as adding, removing or replacing an object) are
also reflected in the `JList`. **Important:** Changing the contents
of a non-observable `List` while it is participating in a
`JListBinding` is unsupported, resulting in undefined behavior and
possible exceptions.

`JListBinding` requires
extra clarification on the operation of the
`refresh` and `save` methods and the meaning of the update
strategy. The target property of a `JListBinding` is not the
target `JList` property provided in the constructor, but rather a
private synthetic property representing the `List` of objects to show
in the target `JList`. This synthetic property is readable/writeable
only when the `JListBinding` is bound and the target `JList`
property is readable with a `non-null` value.

It is this private synthetic property on which the `refresh` and
`save` methods operate; meaning that these methods simply cause syncing
between the value of the source `List` property and the value of the
synthetic target property (representing the `List` to be shown in the
target `JList`). These methods do not, therefore, have anything to do
with refreshing *values* in the `JList`. Likewise, the update
strategy, which simply controls when `refresh` and `save` are
automatically called, also has nothing to do with refreshing *values*
in the `JList`.

**Note:** At the current time, the `READ_WRITE` update strategy
is not useful for `JListBinding`. To prevent unwanted confusion,
`READ_WRITE` is translated to `READ` by `JListBinding's`
constructor.

`JListBinding` works by installing a custom model on the target
`JList`, as appropriate, to represent the source `List`. The
model is installed on a target `JList` with the first succesful call
to `refresh` with that `JList` as the target. Subsequent calls
to `refresh` update the elements in this already-installed model.
The model is uninstalled from a target `JList` when either the
`JListBinding` is unbound or when the target `JList` property
changes to no longer represent that `JList`. Note: When the model is
uninstalled from a `JList`, it is replaced with a `DefaultListModel`,
in order to leave the `JList` functional.

Some of the above is easier to understand with an example. Let's consider
a `JListBinding` (`binding`), with update strategy
`READ`, between a property representing a `List` (`listP`)
and a property representing a `JList` (`jListP`). `listP`
and `jListP` both start off readable, referring to a `non-null`
`List` and `non-null` `JList` respectively. Let's look at
what happens for each of a sequence of events:

| Sequence | Event | Result |
| --- | --- | --- |
| 1 | explicit call to `binding.bind()` | - synthetic target property becomes readable/writeable   - `refresh()` is called   - model is installed on target `JList`, representing list of objects |
| 2 | `listP` changes to a new `List` | - `refresh()` is called   - model is updated with new list of objects |
| 3 | `jListP` changes to a new `JList` | - model is uninstalled from old `JList` |
| 4 | explicit call to `binding.refresh()` | - model is installed on target `JList`, representing list of objects |
| 5 | `listP` changes to a new `List` | - `refresh()` is called   - model is updated with new list of objects |
| 6 | explicit call to `binding.unbind()` | - model is uninstalled from target `JList` |

Notice that in step 3, when the value
of the `JList` property changed, the new `JList` did not
automatically get the model with the elements applied to it. A change to the
target value should not cause an `AutoBinding` to sync the target from
the source. Step 4 forces a sync by explicitly calling `refresh`.
Alternatively, it could be caused by any other action that results
in a `refresh` (for example, the source property changing value, or an
explicit call to `unbind` followed by `bind`).

`DetailBindings` are managed by the `JList`. They are not
to be explicitly bound, unbound, added to a `BindingGroup`, or accessed
in a way that is not allowed for a managed binding.

In addition to binding the elements of a `JList`, it is possible to
bind to the selection of a `JList`. When binding to the selection of a `JList`
backed by a `JListBinding`, the selection is always in terms of elements
from the source `List`, regardless of any `DetailBinding` specified.
See the list of 
interesting swing properties in the package summary for more details.

---

| **Nested Class Summary** | |
| --- | --- |
| `class` | `JListBinding.DetailBinding`             `DetailBinding` represents a binding between a property of the elements in the `JListBinding's` source `List`, and the values shown in the `JList`. |

**Nested classes/interfaces inherited from class org.jdesktop.beansbinding.AutoBinding**

`AutoBinding.UpdateStrategy`

**Nested classes/interfaces inherited from class org.jdesktop.beansbinding.Binding**

`Binding.SyncFailure, Binding.SyncFailureType, Binding.ValueResult<V>`


| **Constructor Summary** | |
| --- | --- |
| `protected` | `JListBinding(AutoBinding.UpdateStrategy strategy, SS sourceObject, Property<SS,java.util.List<E>> sourceListProperty, TS targetObject, Property<TS,? extends javax.swing.JList> targetJListProperty, java.lang.String name)`             Constructs an instance of `JListBinding`. |


| **Method Summary** | |
| --- | --- |
| `protected  void` | `bindImpl()`             Called by `Binding.bind()` to allow subclasses to initiate binding. |
| `JListBinding.DetailBinding` | `getDetailBinding()`             Returns the `DetailBinding` for this `JListBinding`. |
| `JListBinding.DetailBinding` | `setDetailBinding(Property<E,?> detailProperty)`             Creates a `DetailBinding` and sets it as the `DetailBinding` for this `JListBinding`. |
| `JListBinding.DetailBinding` | `setDetailBinding(Property<E,?> detailProperty, java.lang.String name)`             Creates a named `DetailBinding` and sets it as the `DetailBinding` for this `JListBinding`. |
| `protected  void` | `unbindImpl()`             Called by `Binding.unbind()` to allow subclasses to uninitiate binding. |

**Methods inherited from class org.jdesktop.beansbinding.AutoBinding**

`getUpdateStrategy, paramString, sourceChangedImpl, targetChangedImpl`

**Methods inherited from class org.jdesktop.beansbinding.Binding**

`addBindingListener, addPropertyChangeListener, addPropertyChangeListener, bind, bindUnmanaged, firePropertyChange, getBindingListeners, getConverter, getName, getPropertyChangeListeners, getPropertyChangeListeners, getSourceNullValue, getSourceObject, getSourceProperty, getSourceUnreadableValue, getSourceValueForTarget, getTargetNullValue, getTargetObject, getTargetProperty, getTargetValueForSource, getValidator, isBound, isManaged, isSourceUnreadableValueSet, notifySynced, notifySyncFailed, refresh, refreshAndNotify, refreshAndNotifyUnmanaged, refreshUnmanaged, removeBindingListener, removePropertyChangeListener, removePropertyChangeListener, save, saveAndNotify, saveAndNotifyUnmanaged, saveUnmanaged, setConverter, setManaged, setSourceNullValue, setSourceObject, setSourceObjectUnmanaged, setSourceProperty, setSourceUnreadableValue, setTargetNullValue, setTargetObject, setTargetObjectUnmanaged, setTargetProperty, setValidator, throwIfBound, throwIfManaged, throwIfUnbound, toString, unbind, unbindUnmanaged, unsetSourceUnreadableValue`

**Methods inherited from class java.lang.Object**

`clone, equals, finalize, getClass, hashCode, notify, notifyAll, wait, wait, wait`

**Constructor Detail**

### JListBinding

```
protected JListBinding(AutoBinding.UpdateStrategy strategy,
                       SS sourceObject,
                       Property<SS,java.util.List<E>> sourceListProperty,
                       TS targetObject,
                       Property<TS,? extends javax.swing.JList> targetJListProperty,
                       java.lang.String name)
```

:   Constructs an instance of `JListBinding`.

    **Parameters:**: `strategy` - the update strategy: `sourceObject` - the source object: `sourceListProperty` - a property on the source object that resolves to the `List` of elements: `targetObject` - the target object: `targetJListProperty` - a property on the target object that resolves to a `JList`: `name` - a name for the `JListBinding` **Throws:**: `java.lang.IllegalArgumentException` - if the source property or target property is `null`


**Method Detail**

### bindImpl

```
protected void bindImpl()
```

:   **Description copied from class: `Binding`**
:   Called by `Binding.bind()` to allow subclasses to initiate binding.
    Subclasses typically need not install `PropertyStateListeners`
    on the source property and target property as they will be notified
    by calls to `Binding.sourceChangedImpl(org.jdesktop.beansbinding.PropertyStateEvent)` and `Binding.targetChangedImpl(org.jdesktop.beansbinding.PropertyStateEvent)`
    when the source and target properties change respectively.

    :   **Overrides:**: `bindImpl` in class `AutoBinding<SS,java.util.List<E>,TS,java.util.List>`
    :   **See Also:**: `Binding.unbindImpl()`

---


### unbindImpl

```
protected void unbindImpl()
```

:   **Description copied from class: `Binding`**
:   Called by `Binding.unbind()` to allow subclasses to uninitiate binding.

    :   **Overrides:**: `unbindImpl` in class `AutoBinding<SS,java.util.List<E>,TS,java.util.List>`
    :   **See Also:**: `Binding.bindImpl()`

---


### setDetailBinding

```
public JListBinding.DetailBinding setDetailBinding(Property<E,?> detailProperty)
```

:   Creates a `DetailBinding` and sets it as the `DetailBinding`
    for this `JListBinding`. A `DetailBinding` specifies the property
    of the objects in the source `List` to be used as the elements of the
    `JList`. If the `detailProperty` parameter is `null`, the
    `DetailBinding` specifies that the objects themselves be used.

    :   **Parameters:**: `detailProperty` - the property with which to derive each list value from its corresponding object in the source `List` **Returns:**: the `DetailBinding`

---


### setDetailBinding

```
public JListBinding.DetailBinding setDetailBinding(Property<E,?> detailProperty,
                                                   java.lang.String name)
```

:   Creates a named `DetailBinding` and sets it as the `DetailBinding`
    for this `JListBinding`. A `DetailBinding` specifies the property
    of the objects in the source `List` to be used as the elements of the
    `JList`. If the `detailProperty` parameter is `null`, the
    `DetailBinding` specifies that the objects themselves be used.

    :   **Parameters:**: `detailProperty` - the property with which to derive each list value from its corresponding object in the source `List` **Returns:**: the `DetailBinding`

---


### getDetailBinding

```
public JListBinding.DetailBinding getDetailBinding()
```

:   Returns the `DetailBinding` for this `JListBinding`.
    A `DetailBinding` specifies the property of the source `List` elements
    to be used as the elements of the `JList`.

    :   **Returns:**: the `DetailBinding` **See Also:**: `setDetailBinding(Property, String)`


---


|  |  |  |
| --- | --- | --- |
| **Overview** \| **Package** \| **Class** \| **Use** \| **Tree** \| **Deprecated** \| **Index** \| **Help** | |  |
| **PREV CLASS**   **NEXT CLASS** | **FRAMES**    **NO FRAMES**     **All Classes** |  |
| SUMMARY: NESTED \| FIELD \| CONSTR \| METHOD | DETAIL: FIELD \| CONSTR \| METHOD |  |


---
